# Translation direction and word familiarity in word translation: an fNIRS study of intermediate Japanese learners of English

**DOI:** 10.3389/fnhum.2026.1881085

**Published:** 2026-08-05

**Authors:** Wakana Kawai, Kiyomitsu Niioka, Hikari Tanaka, Katsumasa Shinozuka, Yasushi Kyutoku, Ippeita Dan

**Affiliations:** 1 Applied Cognitive Neuroscience Laboratory, Chuo University, Tokyo, Japan; 2 Department of Psychology and Crime, School of Psychological Sciences, University of Human Environments, Ehimei, Japan; 3 Faculty of Global Management, Chuo University, Tokyo, Japan

**Keywords:** functional near-infrared spectroscopy (fNIRS), intermediate L2 learners, Japanese-English learners, revised hierarchical model (RHM), second language, word translation

## Abstract

**Introduction:**

Many studies have examined the neural bases of word translation based on the revised hierarchical model (RHM). However, the findings reported thus far, mostly focusing on advanced or elementary second-language (L2) learners, have been diverse and often inconsistent. Thus, to explore possible neural bases for translation asymmetry, there is a distinct need to examine word translations by intermediate L2 learners.

**Methods:**

We examined differences in cortical activation patterns during L1 (first language)-to-L2 and L2-to-L1word translation for high and low word familiarity in intermediate-level Japanese learners of English. TOEIC scores were used to confirm intermediate English proficiency, and functional near-infrared spectroscopy (fNIRS) was used to examine cortical activation patterns during word translation. Data from 36 participants were used for analyses.

**Results:**

Greater activation was observed in the frontopolar area, the pars triangularis of Broca’s area, and the bilateral dorsolateral prefrontal cortex for low-familiarity words regardless of translation direction.

**Discussion:**

These findings provide no clear evidence of translation asymmetry in intermediate L2 learners but suggest the possible involvement of concept mediation in both translation directions.

## 1 Introduction

With the progress of globalization, many individuals study two or more languages. Particularly in the case of English, which is recognized as an international language, it was estimated more than two decades ago that over 200 million people already spoke it, and that the number would continue to increase thereafter (Crystal, 2008). Further, recent statistics from the European Union revealed that nearly half of the students receiving higher education were studying two or more foreign languages (EUstat). However, given the substantial individual differences in second language acquisition, considerable attention has been focused on understanding the cognitive mechanisms underlying this process and the neural mechanisms involved. In order to elucidate the cognitive mechanisms and neural processes of second language acquisition, studies have investigated multiple linguistic levels, from microscopic units such as phonemes (Callan et al., 2004) to comprehensive units such as discourse (Foucart et al., 2016). Nonetheless, although various results have been reported on the cognitive and neural mechanisms underpinning second language processing, few unified theories have been established. Here, we assumed that among the various linguistic levels, in actual practice, learning words in both languages, which corresponds to word-by-word translation from the first language (L1) to the second language (L2), is essential for L2 acquisition (Schmitt, 2008). Based on this assumption, we considered that clarifying this fundamental process would provide insights applicable to more practical levels of language use, such as phrases, chunks, sentences, and discourse. On this basis, we aimed to elucidate the cognitive mechanisms and neural processes underlying the process of L2 acquisition.

The cognitive process involved in word translation, including translation between languages with different scripts, has been described by the revised hierarchical model (RHM) (Kroll and Stewart, 1994). The RHM assumes that the bilingual mental lexicon consists of mental lexicons of both the L1 and the L2, as well as word concepts shared by both languages. The RHM explains how different cognitive processing occurs depending on the direction of word translation (Kroll et al., 2010; Kroll and Stewart, 1994). Mental lexicons for L1 and L2, and word concept, are connected with links of different strengths. The strength of each link varies depending on L2 proficiency, and the dominant translation route is determined by the relative strength of these links.

For elementary L2 learners, the lexical link from the L2 to the L1 mental lexicons and the bidirectional link connecting the L1 lexicon and word concept are relatively strong, whereas the lexical link from the L1 to the L2 mental lexicons and the bidirectional link connecting the L2 lexicon and word concept are relatively weak. Therefore, the route mediating word concept is dominant in L1-to-L2 translation, and the translation route directly connecting the two lexicons is dominant in L2-to-L1 translation (Schwieter and Sunderman, 2009). As learners become proficient in their L2, the links strengthen, and their dominant route for L1-to-L2 translation changes to the direct link connecting each mental lexicon. This explains the asymmetry in translation, whereby L1-to-L2 translation is more difficult than L2-to-L1 translation, especially for learners with low L2 proficiency (Kroll et al., 2010; Kroll and Stewart, 1994).

Many studies have examined the cognitive processes and neural bases of word translation based on the RHM (Kroll et al., 2010). However, the findings reported thus far have been diverse and often inconsistent. First, the RHM explains that translation asymmetry appears as a function of L2 proficiency (Kroll et al., 2010). From the simplest perspective, if translation asymmetry exists as explained by the model, neural activity patterns should not differ between translation directions for proficient L2 learners, while different neural activity patterns should occur depending on translation direction for low-proficiency learners. However, evidence has been reported both in support of and against the model. One study supporting the model reported that proficient English–French learners exhibited almost no differences in the brain regions activated between translation directions (Klein et al., 1995). Additional similar evidence has been provided by studies involving German–English learners (Price et al., 1999). Other studies contradict that translation asymmetry is dependent on L2 proficiency. Christoffels et al. (2013) reported that proficient Dutch–English learners exhibited larger P2 amplitudes in L1-to-L2 translation and reduced N400 amplitudes in L2-to-L1 translation. Further contradictory results have also been reported; for example, Shinozuka et al. (2021) reported that advanced Japanese–English learners exhibited greater activation in the frontopolar area (FPA) when translating low-familiarity words from L1 into L2 compared with the reverse direction, while elementary learners exhibited no significant differences in cortical activation patterns. Moreover, it has been reported that intermediate- to high-proficiency French–English learners exhibited greater activation 200 ms after the onset of an L1-to-L2 translation task than of an L2-to-L1 translation task, and different cortical regions were activated for 600 ms (Jost et al., 2018). Taken together, these studies suggest that translation asymmetry as a function of L2 proficiency, as implied by the RHM, remains controversial.

Furthermore, based on the RHM, a larger translation asymmetry for low-proficiency learners should occur since low-proficiency learners tend to mediate the word concept during L1-to-L2 translation. This could be interpreted in terms of cortical activation, with increased activation in regions related to semantic processing. It may also involve regions engaged in maintaining and manipulating information to compensate for the higher cognitive load of performing the more complex process of L1-to-L2 translation, compared with L2-to-L1 translation. Broca’s area may be one cortical region that experiences increased activation in L1-to-L2, compared with L2-to-L1, translation, given its well-established role in semantic processing (Demb et al., 1995; Peterson and Savoy, 1998). The FPA, recognized for its role in maintaining (Koechlin and Hyafil, 2007) and integrating (Ramnani and Owen, 2004) multiple tasks, and the right dorsolateral prefrontal cortex (R-DLPFC), implicated in language switching (Fiebach et al., 2002; Hernandez et al., 2001), may exhibit greater activation during L1-to-L2 than L2-to-L1 translation, reflecting the complexity of mediating the concept.

Nonetheless, evidence both supporting and contradicting the RHM and its explained translation asymmetry has been presented. As an example of studies reporting translation asymmetry reflected in some or all of these neural activation patterns, advanced Japanese–English learners exhibited increased activation in the FPA and R-DLPFC, but not in Broca’s area, during L1-to-L2 relative to L2-to-L1 translation (Shinozuka et al., 2021). By contrast, studies on intermediate- to high-proficiency French–English learners reported greater activation in the FPA during L2-to-L1 relative to L1-to-L2 translation (Radman et al., 2018). Moreover, there are studies reporting no translation asymmetry whatsoever. However, the regions activated in these respective studies varied, thereby complicating the interpretation of the cognitive processing underlying word translation. For example, in proficient English–French learners, translation in both directions activated the left ventrolateral and dorsolateral frontal regions, as well as the inferotemporal region (Klein et al., 1995). In contrast, deactivation in the medial superior frontal, left middle temporal, and posterior temporoparietal regions was reported for proficient German–English learners (Price et al., 1999). Thus, when considering translation asymmetry and concept mediation as explained by the RHM, little consistency can be observed across previous studies.

However, this does not in itself invalidate the RHM. Many variables, including L2 proficiency (Perani and Abutalebi, 2005), age of acquisition (AoA), exposure to the L2 (Perani et al., 2003), and others, are known to be related to the neural activity of L2-related tasks, and previous studies have not always accounted for all of these variables. Among these variables, L2 proficiency, which is explicitly described as a variable in the RHM (Kroll et al., 2010), is one of the most significant factors influencing neural activation patterns. Nonetheless, even when accounting for L2 proficiency, inconsistencies remain. Within both groups of proficient and low-proficiency learners, some studies have reported translation asymmetry (Jost et al., 2018; Shinozuka et al., 2021) while others have not (Klein et al., 1995; Price et al., 1999), and the cortical regions reported to be activated also differ. Although other factors may account for the inconsistent results, because L2 proficiency is the only explicit variable in the RHM, we focused on the fact that L2 proficiency itself may be treated inconsistently across these studies. Studies have used their own methods to measure the L2 proficiency of participants, making it difficult to compare proficiency levels across studies. As a result, although learners may have been classified as “proficient,” their actual L2 proficiency levels may have differed significantly. For example, in one study, participants were described as “proficient,” and their L2 proficiency was screened through tasks such as L2 synonym production, L1-to-L2 translation, and giving an oral description of the examination room in their L2 (Klein et al., 1995). In another study, participants were described as “proficient,” and their L2 proficiency was assessed through self-evaluation of their relative proficiency in their L2 (English) compared with their L1 (German) (Price et al., 1999). And again, participants were described as “proficient,” and their L2 proficiency was assessed through a vocabulary test in the form of a non-speeded lexical decision task (Christoffels et al., 2013). Such differences in how proficiency is defined may account for the variability in the results obtained. In other words, among the “proficient” participants in the above studies, some may have had the proficiency that the RHM was designed for, whereas others may not have. Which is to say that participants may not have possessed the same level of L2 proficiency as those in the original RHM study, in which participants exhibited translation asymmetry. Participants in the original study were described as “Dutch–English bilinguals, but not balanced bilinguals,” with their L2 proficiency assessed through a 10-point self-report questionnaire of English proficiency (Kroll and Stewart, 1994), which, it is worth noting, is yet another method of assessing proficiency.

Therefore, to enable valid comparisons across studies, L2 proficiency should be assessed with objectively measurable, widely used, and mutually transferable scoring systems. In order to effectively assess the influence of L2 proficiency on the cognitive processing and neural correlates of word translation, the definition and assessment of language proficiency should be objective and fixed, and more data must be produced based on it. This would make it possible, for the first time, to establish whether hypotheses based on the model hold true or whether the model requires modification in light of additional factors. Previous studies have typically examined only two proficiency groups (high and low), for example, through median split or by selecting participants from the two extremes of proficiency (van Hell and Tanner, 2012). Indeed, the study on brain activity during word translation discussed above also targeted only proficient or low-proficiency learners (i.e., Klein et al., 1995). In our previous study (Shinozuka et al., 2021), we likewise focused on advanced and elementary L2 learners. However, there are learners whose L2 proficiency falls between the two groups. In our previous study and the present study, L2 proficiency was measured using the Test of English for International Communication (TOEIC; The Institute for International Business Communication, 2026a) Listening & Reading (L&R) test. The TOEIC L&R test is an objectively measurable, widely used (approximately 1.78 million people took the test in the Japanese fiscal year 2024; The Institute for International Business Communication, 2026a), and its scores align with measures such as the Common European Framework of Reference for Languages (CEFR) (Tannenbaum and Wylie, 2008). Advanced and elementary learners were defined according to the standards of the test; by focusing on L2 learners who fall outside the range of advanced and elementary groups, which is to say intermediate learners, it should be possible to assess the cortical activation patterns associated with a word translation task and, by incorporating our previous results, compare the patterns at different levels of L2 proficiency. This may help clarify the inconsistent results of previous studies by comparing advanced, intermediate, and elementary learners to identify which patterns are and are not shared.

The purpose of this study was to clarify the differences in cortical activity depending on translation direction in Japanese–English learners with intermediate proficiency. To ensure that the cognitive load was appropriate for intermediate learners, we followed our previous study (Shinozuka et al., 2021) by taking word familiarity into account and establishing high- and low-familiarity conditions. The TOEIC L&R test was used as an objective measurement of L2 English proficiency. To measure cortical activation in a naturalistic environment while performing actual word translation, cortical activity was measured using functional near-infrared spectroscopy (fNIRS). In our previous study (Shinozuka et al., 2021), we showed that, when disregarding their high L2 proficiency, the cortical activation patterns observed in advanced Japanese–English learners during word translation under the low-familiarity condition were consistent with the RHM. In particular, different cortical activation patterns were observed for L1-to-L2 and L2-to-L1 word translation. Additionally, cortical regions such as the FPA and the R-DLPFC, which are assumed to compensate for increased cognitive load, were more greatly activated during L1-to-L2 word translation than during L2-to-L1 translation. If intermediate L2 learners’ cognitive processing is the same as that of advanced learners, similar cortical activation patterns might be observed under the low-familiarity condition. However, considering that differences in word familiarity affect cognitive processing (Connine et al., 1990.; de Groot and Poot, 1997) and that their L2 proficiency is not as high as that of advanced learners, similar patterns may instead be observed under the high-familiarity condition. However, in our previous study (Shinozuka et al., 2021), elementary L2 learners did not exhibit different cortical activation patterns across translation directions, regardless of word familiarity. Thus, if intermediate L2 learners’ cognitive processing is the same as that of elementary learners, there might be little or no difference in cortical activation patterns based on translation direction under either word-familiarity condition.

## 2 Methods

### 2.1 Participants

Initially, 60 undergraduate and graduate students at Chuo University in Japan participated in the study. The TOEIC L&R score was used as a measure of English proficiency due to its relative ease compared to other tests for Japanese students. Participants were required to submit TOEIC L&R scores obtained within 5 weeks before or after the fNIRS measurement. Participants with TOEIC L&R scores between 470 and 725 were defined as intermediate-level English learners. Four advanced learners, 11 elementary learners, and five participants who did not submit TOEIC L&R scores were excluded, and 40 intermediate learners remained. Additional exclusions included two participants who were recognized as left-handed based on the Edinburgh inventory, one participant whose L1 was Chinese, two participants whose AoA was relatively early (approximately 4 and 6 years, respectively), one participant who had lived in the United States for 3 years, and one participant who fell ill during the experiment. The data for the remaining 33 participants were analyzed (mean age: 19.3 ± 1.3, females: 23, males: 10, mean TOEIC L&R score: 557.4 ± 59.2, min: 480, max: 705). Remaining participants had generally started learning English late (AoA: 12.1 ± 0.8 years). The current study was approved by the Institutional Ethics Committee of Chuo University and was in accordance with the latest version of the Helsinki Declaration. Written informed consent was provided from all participants in advance.

### 2.2 Stimuli and experimental procedures

**Figure 1:**
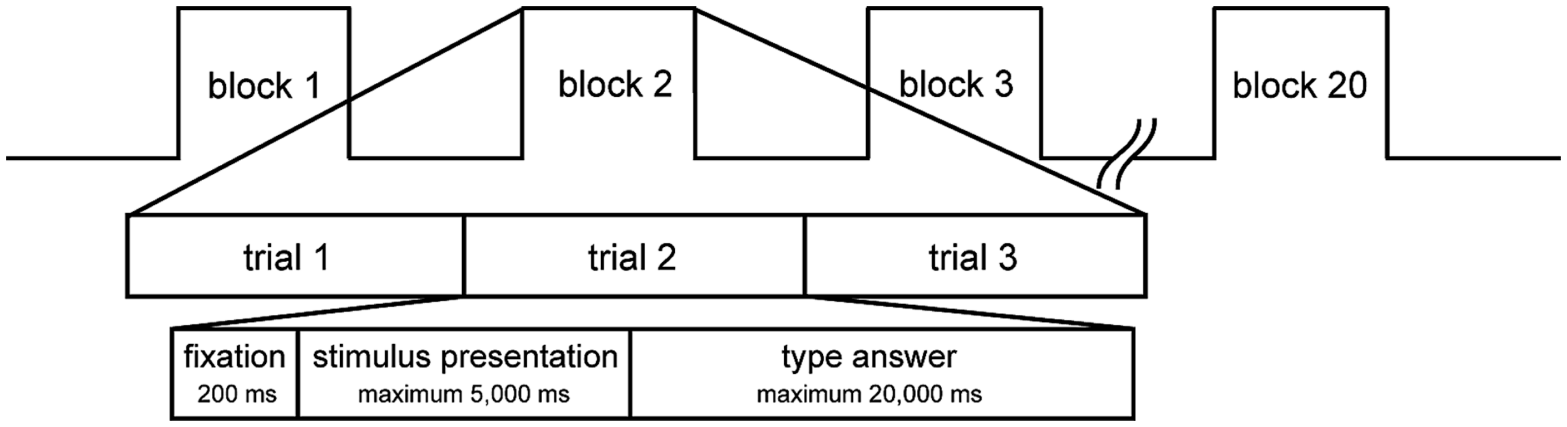
Block design of the word translation task. There were 20 task blocks and one block consisted of three trials. Individual trials started with a 200 ms fixation cross which was followed by a maximum 5,000-ms stimulus presentation. If participants responded, they were given a maximum of 20,000 ms (20 s) of typing time. If they did not respond, the next trial automatically began.

The same word translation task as described in our previous study (Shinozuka et al., 2021) was used here. The task paradigm is illustrated in Figure 1. Participants sat on a chair positioned in front of a monitor. They viewed words displayed on the screen and typed their responses using a keyboard. Upon the presentation of a word, participants were instructed to press the space key if they knew the answer and to press the enter key once they had finished typing their response. When participants could not provide an answer, they were instructed not to press any key. In such cases, the task automatically advanced to the next trial after 5 s. Psychophysics Toolbox, which operates in a Matlab2020b (The Mathworks, Inc., Natick, MA, United States) environment, was used for stimulus presentation and response recording. The task comprised alternating baseline and task blocks, beginning and ending with a baseline block. In the task blocks, participants were required to translate the words, whereas in the baseline blocks, they were instructed to transcribe the words. Task blocks comprised four task conditions based on translation direction (English-to-Japanese and Japanese-to-English) and word familiarity (high and low). There were five task blocks for each of the four conditions, resulting in 20 task blocks in total. Task blocks were presented in random order. All word stimuli were nouns. The number of syllables in English words was set at between one and six, and the number of morae in Japanese words was set at between two and six (Kubozono, 1989). Words were chosen based on word familiarity (Kondo and Amano, 1999; Yokokawa et al., 2007), defined as the average of multiple subjects’ ratings of the degree of familiarity of a word on a 7-point scale for both Japanese and English words. The most familiar 91 words were used as high-familiarity words (e.g., car), and the least familiar 91 words were used as low-familiarity words (e.g., shareholder). In addition, 147 relatively common Japanese words that could be represented using kanji (Chinese characters) alone were selected as baseline words. All Japanese words were presented in kanji, and there were no overlapping words between baseline and task words.

### 2.3 fNIRS data acquisition

**Figure 2:**
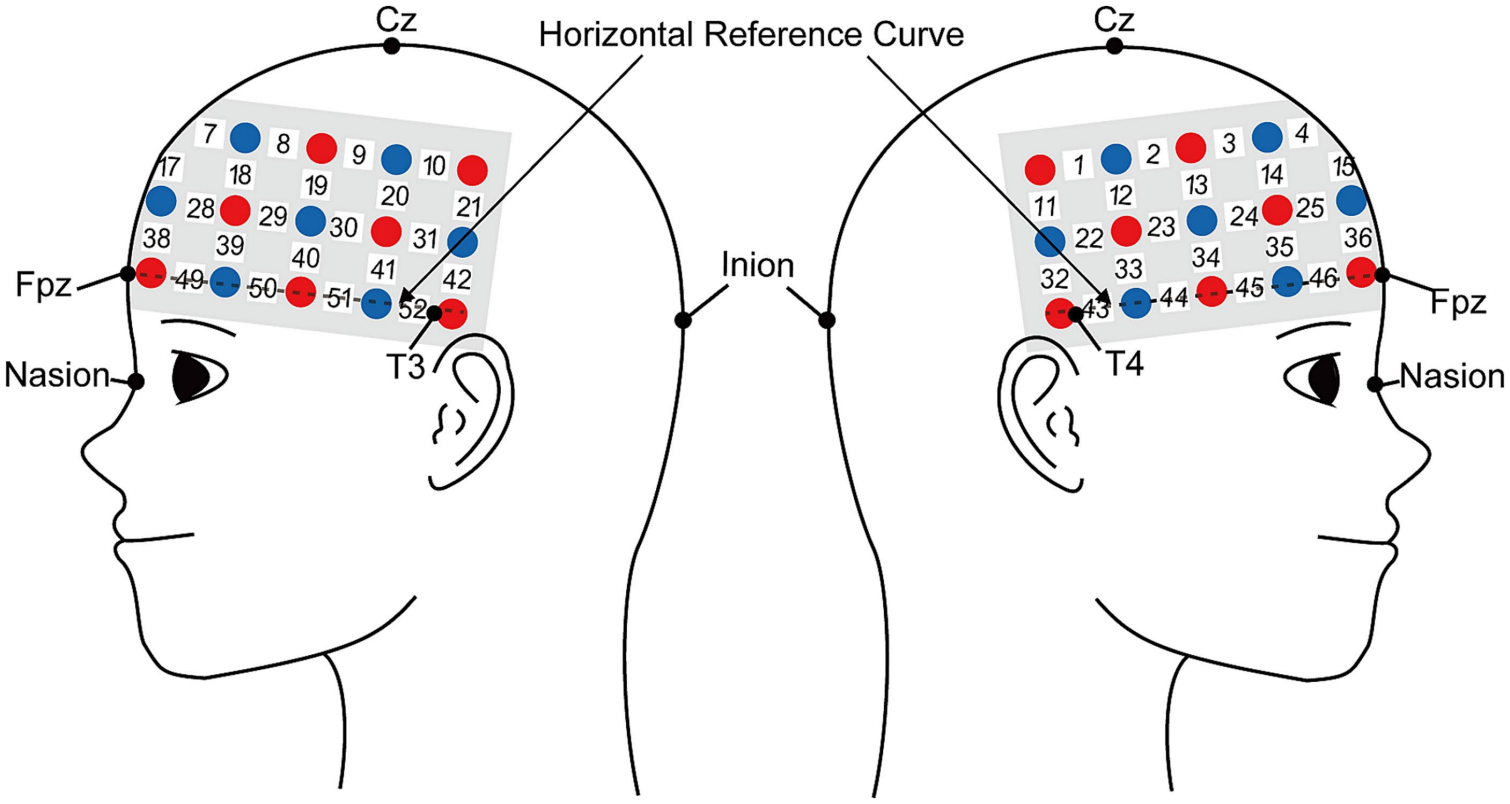
Location of the probe holder. Red circles represent emitters, blue circles represent detectors, and white squares represent channels. Numbers written in the squares are channel numbers.

Data were acquired using an ETG-4000 (Hitachi Medical Corporation, Kashiwa, Japan) CW-NIRS device with 52 active channels (17 laser diode emitters λ_1|2_ = 695|830 nm with average power <4 mW and 16 avalanche photodiode detectors) sampled at 10 Hz. Changes in optical densities were converted into oxygenated hemoglobin (oxy-Hb) and deoxygenated hemoglobin (deoxy-Hb) by applying the modified Beer–Lambert Law (Delpy et al., 1988) and were presented in units of millimolar × millimeter (mM × mm) (Maki et al., 1995). The probe holder was fixed to cover the frontal and temporal areas which are regions related to language processing (Figure 2). Specifically, we set the channel locations according to the 10–20 system of EEG (Jurcak et al., 2007). Detector 26, which is in the center of the 3 × 11 holder, was placed at FPz, and probes on the lowest row were placed so that they were on the line connecting Fpz, T3, and T4 (Figure 2). A 3-D digitizer (POLHEMUS, Patriot) was used to determine the locations of each channel. Anchor-based probabilistic registration (Tsuzuki et al., 2012; Tsuzuki and Dan, 2014) was used for spatial registration to Montreal Neurological Institute (MNI) standard brain space for spatial profiling of the data. The estimated locations were macro-anatomically labeled according to the Brodmann’s atlas of MRIcro (Rorden and Brett, 2000).

### 2.4 fNIRS data analysis

For data preprocessing, first, channels with a signal-to-noise ratio of less than 20 dB were excluded from the analysis as measurement errors. Next, we took the moving average of the data for 5 s. Then, data were pre-whitened using wavelet minimum description length (Jang et al., 2009) and pre-colored by temporal smoothing with convolution of the canonical hemodynamic response function (cHRF) to the individual time series data (Friston et al., 2000a, 2000b). Finally, effects of extracerebral hemodynamics were reduced using the optimized PCA approach (Kawai et al., 2026). The optimal CSU was selected as 1.9 for oxy-Hb and 1.0. As a first-level analysis, the data were further processed using a general linear model (GLM) analysis. The cHRF was used as the basis function (Friston et al., 2000a, 2000b) and was generated by convolving the HRF with a boxcar function. We took an adaptive GLM approach, adjusting the first peak (τ_p_) of the HRF (Uga et al., 2014). We explored optimal τ_p_ from 6 s to 25 s. The optimal τ_p_, which was 20 s for both oxy- and deoxy-Hb, was set to maximize the average R^2^ of all participants. We used default values for the second peak of the cHRF (τ_d_), 10 s, and an amplitude (A) of 6. The baseline condition, each task condition, and the first and second derivatives of each condition representing individual noise and the constant were included in the regressor. The *β* values representing the degree of activation for each task condition were used in the following analyses. For second-level analysis, we first conducted one-sample *t*-tests (vs. 0, *p* = 0.05, two-tailed) on *β* values in each condition to find activated regions in order to set our regions of interest (ROI). The adjusted *M*_eff_ method (Yamamoto et al., 2024) was used for family-wise error correction. The determined *M*_eff_ values are listed in Supplementary Table 1. We then performed a 2 × 2 repeated-measures ANOVA (two-tailed) on ROIs. The Bonferroni correction was used for family-wise error correction.

### 2.5 Behavioral data analysis

Reaction times (RTs; ms) were defined as starting at stimulus presentation to the time at which the space key was pressed. The average of all RTs for correctly translated words was calculated. Regarding calculations for accuracy (ACC), a correct answer was a word in the opposite language with the same meaning as the stimulus as defined in either the Online Cambridge Dictionary (Cambridge Dictionary | English Dictionary, Translations and Thesaurus, 2026) the OLEX English-Japanese Dictionary (Nomura et al., 2016), or the Genius English-Japanese Dictionary (Konishi and Minamide, 2001). Answers were checked by two authors (WK and HT) after being automatically scored using model answers. Answers in both upper and lower cases were considered correct. One mistake in a word was considered a typing mistake and a word with two or more mistakes was considered incorrect. One mistake was calculated according to Levenshtein distance implemented in Matlab. However, mistakes in numbers (e.g., ‘October’ to ‘*9 gatsu*’, which should be ‘*10 gatsu*’) were not considered typing mistakes even if they only contained one mistake within a word. IBM SPSS Statistics version 28 (IBM) was used for further analysis of behavioral data. A two-way repeated measures ANOVA was employed to investigate the main and interaction effects of translation direction and word familiarity on RT and ACC.

## 3 Results

### 3.1 Exploratory fNIRS analysis for setting ROIs

**Figure 3:**
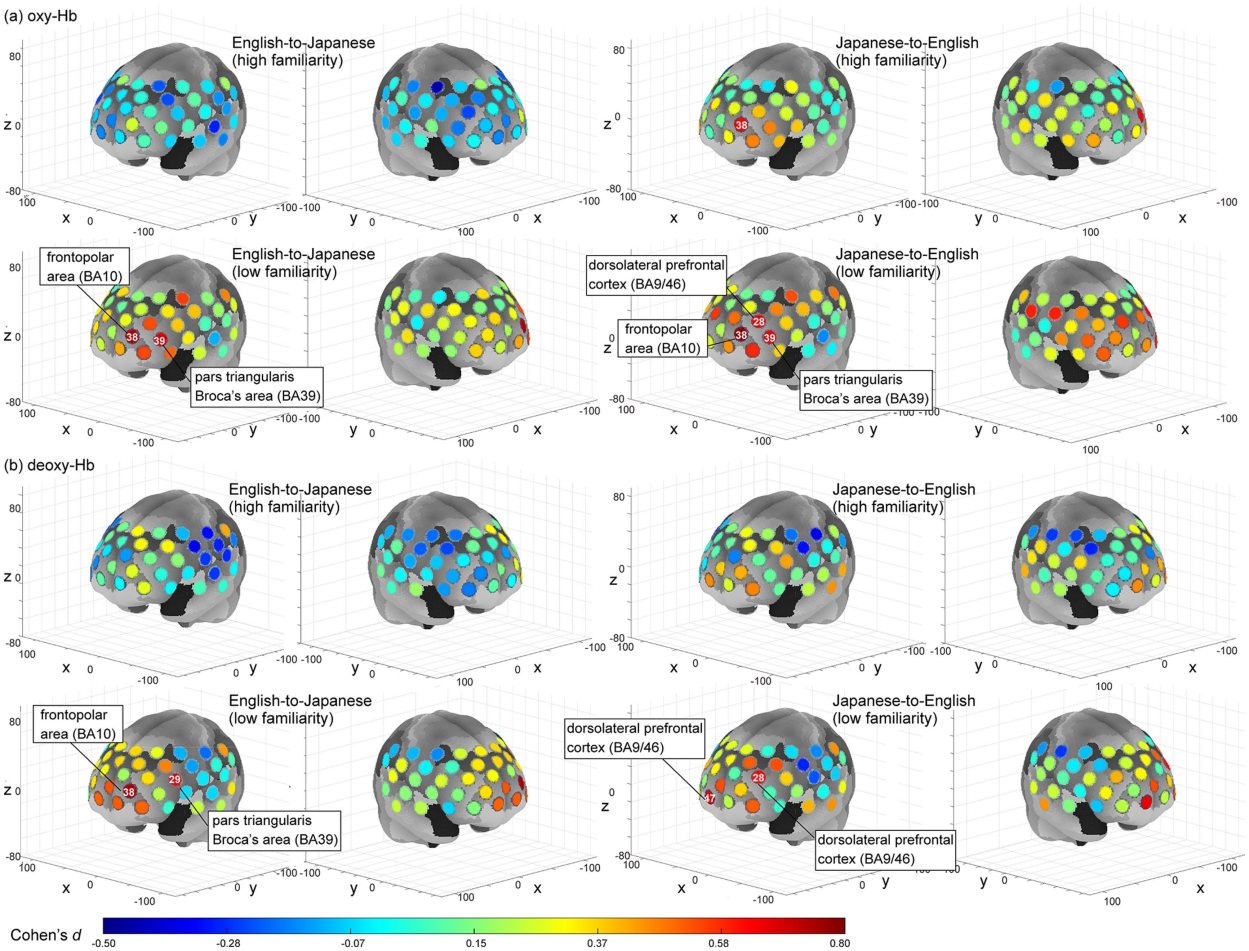
Size of cortical activation in each task condition. Channel numbers of significantly activated channels are shown in white and the corresponding region name is indicated. The estimated channel locations are shown on the standard brain in MNI space. **(a)** oxy-Hb, **(b)** deoxy-Hb.

The cortical regions of the channels (CHs) that were significantly activated for each task condition are displayed in Figure 3. In summary, three regions were determined as ROIs for oxy-Hb: the L-DLPFC (BA46; CH28), the FPA (BA10; CH38), and the pars triangularis Broca’s area (BA45; CH39). For deoxy-Hb, four regions were determined as ROIs: the L-DLPFC (BA46, CH28), the pars triangularis Broca’s area (BA45; CH29), the FPA (BA10; CH38), and the R-DLPFC (BA46; CH47). The most likely estimated regions and their MNI coordinates for all CHs are shown in Supplementary Table 2. Results of all one-sample *t*-tests are shown in Supplementary Table 3 for oxy-Hb and Supplementary Table 4 for deoxy-Hb. Block-averaged waveforms of each task condition are shown in Supplementary Figures 1–4.

### 3.2 ROI-based fNIRS analyses of translation direction and word familiarity

**Figure 4:**
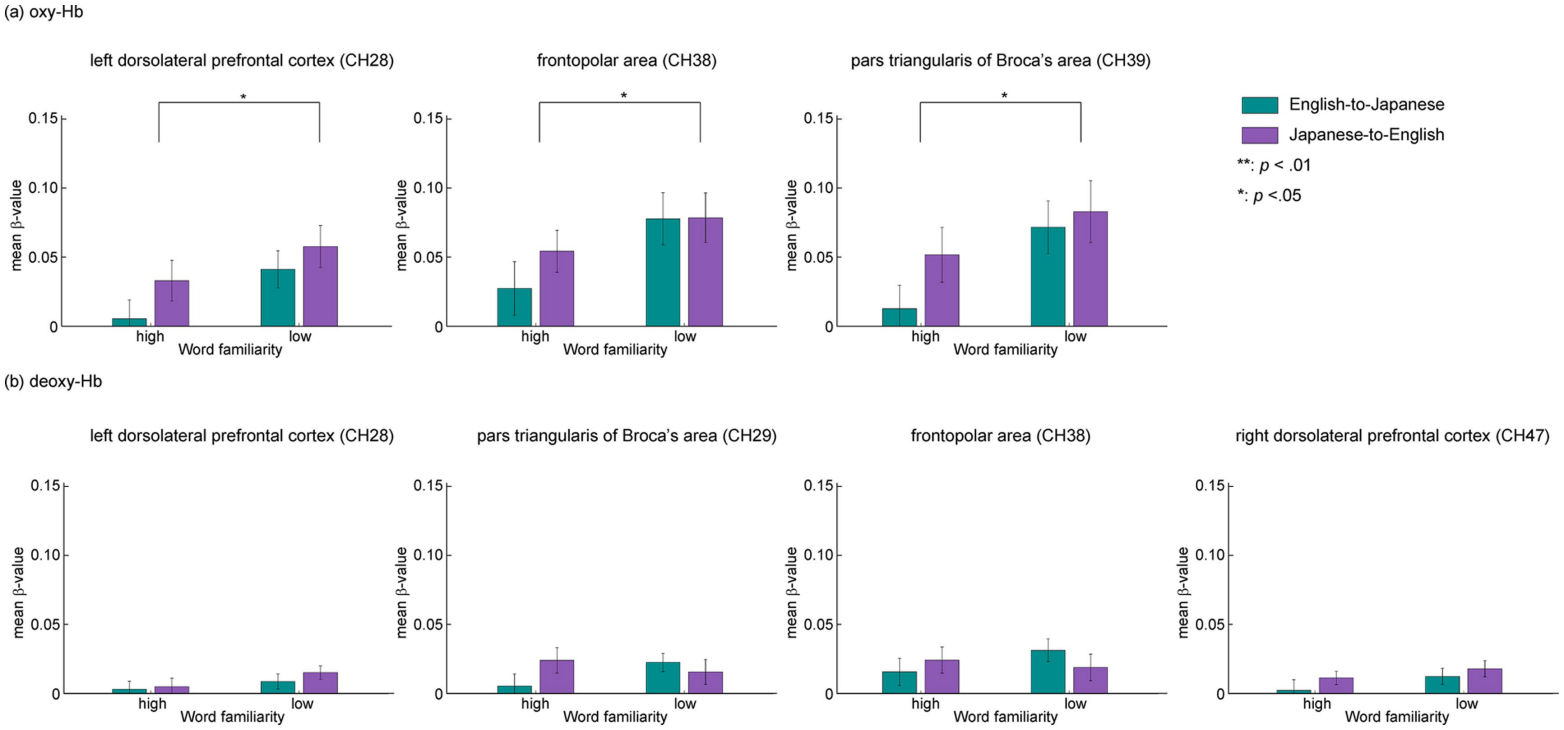
Results of two-way ANOVAs on translation direction and word familiarity for each ROIs. Error bars represent SE. **(a)** oxy-Hb, **(b)** deoxy-Hb.

Overall, two-way ANOVAs examining the effects of translation direction and word familiarity on oxy-Hb cortical activation across all ROIs revealed significant main effects of word familiarity in the L-DLPFC: *F* (1,31) = 7.88, *p <* 0.01, $\eta_{p}^{2}$ = 0.21, the FPA: *F* (1,31) = 12.2, *p <* 0.01, $\eta_{p}^{2}$ = 0.24, and the pars triangularis of Broca’s area: *F* (1,30) = 9.62, *p <* 0.01, $\eta_{p}^{2}$ = 0.26 (Figure 4). No significant main effects of translation direction or interactions between word familiarity and translation direction were detected. In the deoxy-Hb analysis, no significant main effects of word familiarity, translation direction, or interaction between word familiarity and translation direction were detected.

### 3.3 Results from behavioral data analyses

A two-way repeated measures ANOVA was employed to investigate the main and interaction effects of translation direction and word familiarity on RT. The main effect of word familiarity was significant: *F* (1,27) = 51.2, *p <* 0.001, $\eta_{p}^{2}$ = 0.66. No significant main effects of translation direction were detected. The interaction effect of translation direction and word familiarity was also significant: *F* (1,27) = 6.16, *p <* 0.05, $\eta_{p}^{2}$ = 0.19. All pairwise comparisons among means were tested using a Bonferroni correction at *α* = 0.05. A simple main effects analysis revealed shorter RTs for the L2-to-L1 direction than for the L1-to-L2 direction with high-familiarity words. Words with high familiarity had shorter RTs than low-familiarity words, regardless of translation direction.

Similarly, a two-way repeated measures ANOVA was employed to investigate the main and interaction effects of translation direction and word familiarity on ACC. The main effect of word familiarity was significant: *F* (1,32) = 1,216, *p <* 0.001, $\eta_{p}^{2}$ = 0.97. The main effect of translation direction was also significant, *F* (1,32) = 32.3, *p <* 0.001, $\eta_{p}^{2}$ = 0.50. The interaction effect of translation direction and word familiarity was also significant: *F* (1,32) = 12.5, *p <* 0.01, $\eta_{p}^{2}$ = 0.28. All pairwise comparisons among means were tested using a Bonferroni correction at *α* = 0.05. A simple main effects analysis revealed higher ACC for the L2-to-L1 direction than for the L1-to-L2 direction in high-familiarity words. Words with high familiarity had a higher ACC than low-familiarity words, regardless of translation direction. Detailed statistical results of the simple main effects analysis for RT and ACC are shown in Table 1.

**Table 1:** Interaction of translation direction and word familiarity.

Behavioral measure	Word familiarity	English to Japanese		Japanese to English	
		*M*	*SE*	*M*	*SE*
RT	High familiarity	1.54_a_	0.05	1.71_b_	0.08
	Low familiarity	2.3_c_	0.12	2.09_c_	0.14
ACC	High familiarity	0.90_a_	0.02	0.73_b_	0.02
	Low familiarity	0.23_c_	0.02	0.17_d_	0.02

Means with different subscripts indicate *p <* 0.05 in Bonferroni correction. RT, reaction time; ACC, accuracy.

## 4 Discussion

### 4.1 Summary of the study

The neural bases of word translation have often been discussed within the framework of the RHM. The RHM explains translation asymmetry as a function of L2 proficiency, with asymmetry diminishing as learners become more proficient in their L2 (Kroll et al., 2010). In this model, translation asymmetry is assumed to occur because L1-to-L2 translation imposes greater cognitive load, as it tends to involve mediation by word concept, whereas L2-to-L1 translation can rely more on a direct route between the L1 and L2 mental lexicons that does not involve mediation by word concept. However, previous findings based on the RHM, most of which have focused on advanced or elementary L2 learners, have been diverse and often inconsistent. The purpose of this study was to clarify the differences in cortical activity depending on translation direction in Japanese–English learners with intermediate proficiency. To ensure that the cognitive load was appropriate for intermediate learners, we followed the methodology of our previous study (Shinozuka et al., 2021) by taking word familiarity into account and establishing high- and low-familiarity conditions. Cortical activation was measured using fNIRS. Since it was unclear which cortical regions would activate in intermediate learners, we first conducted statistical analyses to determine the ROIs. Three ROIs were identified for oxy-Hb: the L-DLPFC (BA46), the FPA (BA10), and the pars triangularis of Broca’s area (BA45). For deoxy-Hb, four ROIs were identified: the L-DLPFC (BA46), the pars triangularis Broca’s area (BA45), the FPA (BA10), and the R-DLPFC (BA46). Subsequently, two-way ANOVAs were performed for each ROI separately for oxy-Hb and deoxy-Hb to examine the main and interaction effects of translation direction and word familiarity on cortical activation. The ANOVAs revealed significant main effects of word familiarity in the L-DLPFC, FPA, and the pars triangularis of Broca’s area for oxy-Hb. No significant main effects of translation direction or interactions between translation direction and word familiarity were observed. There were no significant main effects or interaction effects for deoxy-Hb. In the following sections, we first interpret the results obtained from intermediate learners in relation to translation asymmetry and concept mediation as explained by the RHM. We then discuss the present findings with particular attention to L2 proficiency by comparing them with previous studies focusing on advanced or elementary L2 learners.

### 4.2 Intermediate learners

For the intermediate learners examined in the present study, the results can be interpreted as providing no clear evidence of translation asymmetry. If translation asymmetry were present, cortical activation patterns would be expected to differ depending on translation direction. However, no significant effects of translation direction were observed in the ROIs defined in the present study. These findings therefore suggest that translation asymmetry was not clearly observed in intermediate Japanese learners of English.

On the other hand, with respect to concept mediation, the present results may suggest that intermediate learners relied on a neural route mediating word concept during word translation, regardless of translation direction. Significant activation was observed in the L-DLPFC, pars triangularis of Broca’s area and the FPA for oxy-Hb, and in the FPA, pars triangularis of Broca’s area, and bilateral DLPFC for deoxy-Hb. All significantly activated regions for oxy-Hb showed significantly greater activation in the low-familiarity condition than in the high-familiarity condition. The absence of significant differences between task conditions in deoxy-Hb may be attributable to the lower sensitivity of deoxy-Hb to cognitive processes compared with that of oxy-Hb (Hoshi, 2003). Broca’s area has been associated with semantic processing (Demb et al., 1995; Peterson and Savoy, 1998), whereas the FPA is involved in maintaining (Koechlin and Hyafil, 2007) and integrating (Ramnani and Owen, 2004) multiple tasks. In addition, the L-DLPFC is known to be involved in task preparation and execution in bilinguals (Seo et al., 2018). Taken together, activation in these regions may suggest word concept mediation during word translation of low-familiarity words by intermediate learners.

### 4.3 Translation asymmetry, concept mediation, and L2 proficiency

When the present findings for intermediate L2 learners are compared with those for the elementary and advanced learners in our previous study (Shinozuka et al., 2021), intermediate learners appear to show a pattern similar to that of elementary learners with respect to translation asymmetry, but a pattern similar to that of advanced learners with respect to concept mediation. Translation asymmetry has been considered to vary as a function of L2 proficiency, with asymmetry diminishing as learners become more proficient in their L2. According to this, intermediate learners would be expected to exhibit translation asymmetry; however, no clear evidence of translation asymmetry was observed in the present study. These findings may be interpreted as indicating that intermediate learners are more similar to elementary learners, who show no translation asymmetry regardless of word familiarity, than to advanced learners, who show translation asymmetry in the low-familiarity condition. When the findings of our previous study (Shinozuka et al., 2021) are integrated with those of the present study, it becomes clear that the relationship between L2 proficiency and translation asymmetry may not be linear. Rather, elementary and intermediate learners may show little or no translation asymmetry, whereas advanced learners may show translation asymmetry when task demands are high, such as in the low-familiarity condition, but not when task demands are low such as in the high-familiarity condition. Taken together, these findings raise the possibility that the occurrence of translation asymmetry is not explained by a simple linear function of L2 proficiency, but instead follows a more complex, potentially inverted U-shaped relationship in which task difficulty also plays an important role.

Further, we can consider the present results and the above interpretation with other previous studies. For studies in which translation asymmetry was not observed, two possibilities can be considered: the participants may in fact have been low or intermediate L2 learners, or they may have been advanced learners for whom the words used in the translation task were relatively easy. Proficient French–English and German–English advanced learners in whom no translation asymmetry was observed indeed fall into the latter category (Klein et al., 1995; Price et al., 1999). However, even within this narrowed categorization, there are differences. In Klein et al., activation was observed in the left ventrolateral and dorsolateral frontal regions as well as the inferotemporal region, whereas in Price et al., deactivation in the medial superior frontal, left middle temporal and posterior temporoparietal regions was reported. Considering significantly activated cortical regions, the results from Klein et al. are relatively close compared to the results from Price et al., indicating that participants of Klein et al. may have actually been intermediate L2 learners. However, considering that the significantly activated regions are not exactly the same, and further considering different L2 pairs, different measurement modalities, and different experimental designs, we should be aware that this interpretation of participant level suggests only one potential explanation for the variation in their results.

For studies in which translation asymmetry was observed, they may have been advanced learners for whom the words used in the translation task were relatively difficult. Intermediate to highly proficient French–English learners and advanced German–English learners from several previous studies fall into this category (Christoffels et al., 2013; Jost et al., 2018; Radman et al., 2018). These studies investigated event-related potentials (ERPs) rather than cortical functions, making direct comparison with the present findings difficult. Nevertheless, different tendencies suggesting translation asymmetry were also reported within these studies.

Thus, although L2 proficiency and relative task difficulty may offer a partial explanation for translation asymmetry, they do not fully account for the observed differences in activation patterns. Furthermore, the intermediate learners in the present study showed significantly longer RTs and lower ACC in the L1-to-L2 translation of low-familiarity words, which appears inconsistent with the fNIRS results showing no clear effect of translation direction. Although most previous studies did not report such discrepancies between behavioral and neural results (Christoffels et al., 2013; Klein et al., 1995; Jost et al., 2018; Price et al., 1999), similar discrepancies were also observed in our previous study (Shinozuka et al., 2021). Given that the most notable difference between our studies and other previous studies is the response modality, typing responses using a keyboard may be one factor contributing to this discrepancy. These findings suggest the need to investigate additional variables that may also play a role.

With respect to concept mediation, the significantly activated regions observed in the intermediate learners in the present study may be interpreted as suggesting that they relied on the word concept in the low-familiarity condition, regardless of translation direction. This pattern was more similar to that observed in advanced learners in our previous study (Shinozuka et al., 2021), who showed evidence suggesting concept mediation during L1-to-L2 translation of low-familiarity words, than to that observed in elementary learners, who did not show activation in regions related to concept mediation. According to the RHM, translation asymmetry occurs because the route mediated by the word concept is more dominant in L1-to-L2 word translation, whereas the direct route between L1 and L2 mental lexicons, which does not involve word concept mediation, is more dominant in L2-to-L1 word translation. The regions significantly activated in intermediate learners, namely the FPA, the pars triangularis of Broca’s area, and the bilateral DLPFC, largely overlapped with those significantly activated in advanced learners. The only region that showed significant activation only in intermediate learners was the L-DLPFC. The L-DLPFC has been reported to show increased activation when language proficiency is insufficient during second-language use (Abutalebi and Green, 2007). Given that the low-familiarity condition was interpreted as imposing a high cognitive load on advanced learners, this condition may have imposed an even greater cognitive load on intermediate learners. This interpretation is further supported by behavioral results, revealing significantly longer RTs and lower ACC for the low familiarity condition. This raises the possibility that concept mediation in intermediate learners is less efficient or less fully developed than that in advanced learners. In summary, the present results suggest that intermediate learners may rely on word concept in a manner similar to advanced learners, but that this reliance may be incomplete or less efficient.

### 4.4 Limitations and future perspectives

The findings of the current study should be interpreted in light of the following limitations. First, we assumed the RHM, which has been criticized for its inconsistent results regarding translation asymmetry (Brysbaert and Duyck, 2010), as the cognitive model for word translation. Since its initial proposal, an extended version of the RHM that incorporates control of the two languages has been put forward (Green, 1998), but there have been no further updates. In its place, we could have considered utilizing an alternative framework, the Bilingual Interactive Activation Model (BIA; Dijkstra et al., 1998), originally developed to account for bilingual word recognition, together with its extended version adapted for word translation (Dijkstra et al., 2019). However, models derived from the BIA are based on the assumption that the two languages involved in word translation share the same script (i.e., the Roman alphabet). The present study involved translation between English, which employs an alphabetic script, and Japanese, which employs logographic and syllabic scripts. For that reason, we opted for the RHM.

Second, we considered L2 proficiency measured using the TOEIC L&R test as a variable. This test does not directly measure the productive L2 abilities required for the present word translation task, but rather assesses broad English communication abilities (The Institute for International Business Communication, 2026b). Therefore, the term “intermediate learners” in the present study should be interpreted as referring to learners with intermediate-level broad English communication abilities and does not necessarily indicate intermediate-level productive English abilities. Furthermore, as noted above, beyond L2 proficiency (Perani and Abutalebi, 2005), AoA, and exposure to the target language (Perani et al., 2003) are also regarded as factors influencing the neural processing involved in L2 use. However, rather than control for these factors, we excluded participants who may be considered extreme outliers. This approach was adopted to ensure a sufficient number of participants could be enrolled in the study. While controlling for all possible factors to isolate the effect of L2 proficiency is impractical, future studies should consider which fundamental factors are most relevant and influential.

Third, the participants in the present study had Japanese as their L1 and English as their L2. Different L1–L2 pairs are one example of a variable that affects the outcomes of L2 tasks. In particular, the possibility that different cognitive processing may occur between bilinguals of same-script and different-script languages has been pointed out (Sunderman and Priya, 2012). Indeed, different cortical activation patterns have been observed in association with different cognitive processes (Kim et al., 2016). Regarding the effects of different L1–L2 pairs (Christoffels et al., 2013; Klein et al., 1995; Price et al., 1999; Shinozuka et al., 2021) no previous studies have examined the exact same pairings, which may have resulted in different findings between similar studies. Thus, the results of the present study may be limited to individuals whose L1 is Japanese and L2 is English. Future studies should investigate whether similar results can be obtained using different L1–L2 pairs.

Fourth, the participants recruited in this study were young adults. Age may be another factor influencing performance in word translation. In the translation task, participants were required to maintain the word in one language, retrieve its equivalent in the other language, and produce the translated word. These processes are likely to involve domain-general executive functions. Previous studies have shown that domain-general executive functions reach their peak in late adolescence or young adulthood (Tervo-Clemmens et al., 2023) and decline in older adulthood (Filippi et al., 2020). Therefore, the present findings obtained from intermediate learners may be specific to young adults. Future studies should examine the effects of age by recruiting younger adolescents or older adults with comparable intermediate English proficiency.

Fifth, the present study focused on studies employing the translation production paradigm. For example, the translation recognition task (De Groot, 1992) is an experimental paradigm commonly used to examine the cognitive processing of word translation. The translation recognition task differs from actual translation production, where translation recognition paradigms require the consistent selection of identical translation pairs. Thus, there is a possibility that translation production and translation recognition require different cognitive processes. Further, some studies have adopted experimental paradigms using sentences rather than single words (Elmer, 2016; He et al., 2017; Hervais-Adelman et al., 2015; Zheng et al., 2020). This paradigm differs from simple word translation in that it incorporates both syntactic and contextual information. While we chose to focus exclusively on studies employing the translation production paradigm, since some previous studies have treated these paradigms inclusively (He et al., 2021; Quaresima et al., 2002), future research should also consider whether they should be treated in this way in order to develop a comprehensive understanding of both single-word and sentence translation.

Sixth, in the present study, word translation was performed by typing on a keyboard. However, we did not consider the effect of typing proficiency, which might have influenced the cortical activation and behavioral results. This may have introduced the discrepancies between behavioral data, which showed translation asymmetry for translating low-familiarity words, and cortical activation, which exhibited no translation asymmetry. The effect of typing proficiency cannot be completely excluded since typing proficiency was not assessed in the present study or in our previous study (Shinozuka et al., 2021). Future studies should take typing proficiency into account and control for its potential effects by treating it as a covariate.

Finally, the ROIs used to test the effects of translation direction and word familiarity were defined based on channels that showed significant activation in the one-sample *t*-tests. While this approach helps ensure that differences across task conditions are interpreted within regions showing task-related responses, it may overlook significant translation-direction-related differences in channels that do not specifically exhibit significant task-related activation. Furthermore, although this approach has traditionally been used in fMRI and fNIRS studies, it entails the risk of “double dipping,” which can inflate the likelihood of Type I errors (Kriegeskorte et al., 2009). However, because the present study was exploratory, the ROIs could not be specified *a priori*. In addition, recruiting a large number of participants was infeasible. We therefore adopted this conventional approach. To address this limitation, we provide descriptive statistics and the results of one-sample *t*-tests for all CHs in each task condition in Supplementary Tables 2, 3, and 4, so that these data can be used in future synthetic studies.

## 5 Conclusion

In conclusion, this study examined cortical activation during L1-to-L2 and L2-to-L1 word translation in intermediate Japanese learners of English using high- and low-familiarity word conditions. fNIRS measurements revealed significantly greater activation in the low-familiarity condition in the L-DLPFC, FPA, and the pars triangularis of Broca’s area for oxy-Hb. In contrast, no significant main effect of translation direction or interaction between translation direction and word familiarity was observed. These findings provide no clear evidence of translation asymmetry in intermediate L2 learners, but suggest the possible involvement of concept mediation, particularly during low-familiarity word translation, regardless of translation direction. Together with previous findings from elementary and advanced L2 learners, the present results suggest that the occurrence of translation asymmetry may depend on L2 proficiency and the relative task difficulty with respect to the learner’s proficiency. However, these factors alone do not fully explain the diverse activation patterns reported in previous studies. Further research is therefore needed to identify additional variables involved in word translation and to refine the RHM as a framework for explaining the cognitive processes underlying bilingual word translation. Regarding practical applications, by focusing on word-level translation and observing different cortical activation patterns dependent upon L2 proficiency, this study provides a possible foundation for the development of neuroimaging-based assessments of L2 learners’ proficiency. Moreover, while learning a second language is, indeed, carried out by evolving brain functions involved in language processing, most learners do not realize what is going on in their brains. One novel potential of this strategy is to use imaging tools such as fNIRS to help learners visualize changes in their own cortical activation patterns as they learn, which could, in turn, help them improve their proficiency level–an innovative approach which is currently beyond the reach of traditional educational practices, providing a technical basis for neuroimaging-based education to come.

## Data Availability

The raw data supporting the conclusions of this article will be made available by the authors, without undue reservation.
