# Supplementary material for: Translation direction and word familiarity in word translation: an fNIRS study of intermediate Japanese learners of English
Source: Front Hum Neurosci. 2026 Aug 5;20:1881085. doi: 10.3389/fnhum.2026.1881085 (PMC13485910; doi:10.3389/fnhum.2026.1881085)
Supplement: Supplementary file 1 [file Supplementary_file_1.docx]

Supplementary Material

# 1 Supplementary Figures and Tables

**Supplementary Table 1.** M_eff_ values for both oxy-Hb and deoxy-Hb for each task condition.

|  | English-into-Japanese | Japanese-into-English |  |  | English-into-Japanese | Japanese-into-English |
| --- | --- | --- | --- | --- | --- | --- |
| high familiarity | 24.50 | 24.44 |  | high familiarity | 28.47 | 26.47 |
| low familiarity | 22.68 | 23.84 |  | low familiarity | 24.52 | 28.03 |

**Supplementary Table 2**. Most likely estimated regions and their MNI coordinates

|  | MNI coordinates | | | SD | Brodmann's area |  |
| --- | --- | --- | --- | --- | --- | --- |
| CH | x | y | z | (mm) |  | % |
| 1 | 62.7 | -40.7 | 50.3 | 11.6 | R-SMG (BA40) | 96.9 |
| 2 | 60.0 | -13.7 | 51.3 | 10.8 | R-PMC (BA4) | 39.3 |
| 3 | 49.0 | 12.3 | 53.3 | 9.9 | R-DLPFC (BA9/46) | 59.6 |
| 4 | 32.3 | 31.3 | 54.0 | 9.9 | R-DLPFC (BA9/46) | 49.7 |
| 5 | 12.3 | 42.7 | 55.3 | 9.5 | Includes FEF (BA8) | 52.6 |
| 6 | -10.7 | 42.7 | 55.0 | 9.4 | Includes FEF (BA8) | 51.9 |
| 7 | -30.7 | 29.7 | 54.0 | 9.9 | Includes FEF (BA8) | 55.0 |
| 8 | -47.3 | 10.3 | 53.3 | 10.0 | L-PMC and SMA (BA6) | 59.2 |
| 9 | -58.7 | -16.7 | 50.7 | 10.9 | L-S1 (BA2/3) | 47.9 |
| 10 | -61.0 | -42.7 | 49.7 | 11.6 | L-SMG; Wernicke's area (BA40) | 100.0 |
| 11 | 64.0 | -51.3 | 37.7 | 12.6 | L-SMG; Wernicke's area (BA40) | 60.2 |
| 12 | 69.0 | -22.7 | 38.3 | 11.1 | R-S1 (BA2/3) | 53.1 |
| 13 | 61.0 | 6.3 | 39.3 | 10.0 | R-PMC and SMA (BA6) | 76.2 |
| 14 | 46.7 | 32.7 | 41.7 | 9.1 | R-DLPFC (BA9/46) | 41.6 |
| 15 | 24.7 | 50.7 | 43.7 | 9.4 | R-DLPFC (BA9/46) | 98.0 |
| 16 | 0.0 | 54.0 | 42.3 | 9.3 | R-DLPFC (BA9/46) | 100.0 |
| 17 | -22.3 | 49.3 | 43.3 | 9.9 | L-DLPFC (BA9/46) | 98.1 |
| 18 | -44.7 | 30.7 | 41.7 | 9.6 | L-DLPFC (BA9/46) | 40.2 |
| 19 | -59.0 | 3.7 | 39.7 | 10.6 | L-PMC and SMA (BA6) | 71.6 |
| 20 | -67.0 | -25.7 | 37.3 | 11.0 | L-S1 (BA2/3) | 65.0 |
| 21 | -62.3 | -53.3 | 37.3 | 12.0 | L-SMG; Wernicke's area (BA40) | 55.9 |
| 22 | 71.0 | -34.3 | 24.7 | 12.4 | L-STG (BA22) | 39.0 |
| 23 | 68.3 | -2.3 | 25.7 | 10.2 | R-SCA (BA43) | 83.3 |
| 24 | 57.0 | 27.3 | 27.7 | 9.4 | R-PTr; Broca's area (BA45) | 73.1 |
| 25 | 39.7 | 50.7 | 30.7 | 8.8 | R-DLPFC (BA9/46) | 90.2 |
| 26 | 15.3 | 64.0 | 32.3 | 8.7 | FPA (BA10) | 67.0 |
| 27 | -13.3 | 63.3 | 32.3 | 8.9 | FPA (BA10) | 61.7 |
| 28 | -37.3 | 50.3 | 30.3 | 9.4 | L-DLPFC (BA9/46) | 89.8 |
| 29 | -54.7 | 25.7 | 27.7 | 9.8 | L-PTr (BA45) | 61.8 |
| 30 | -66.7 | -5.7 | 25.7 | 10.3 | L-SCA (BA43) | 94.2 |
| 31 | -69.0 | -36.7 | 24.3 | 12.0 | L-STG (BA22) | 41.1 |
| 32 | 71.0 | -45.3 | 7.7 | 12.7 | L-STG (BA22) | 57.5 |
| 33 | 71.0 | -14.3 | 8.7 | 11.5 | R-STG (BA22) | 84.4 |
| 34 | 63.0 | 18.3 | 13.3 | 9.9 | R-POp (BA44) | 42.5 |
| 35 | 51.3 | 46.7 | 14.7 | 8.8 | R-PTr (BA45) | 52.7 |
| 36 | 29.0 | 66.7 | 17.0 | 8.8 | FPA (BA10) | 95.5 |
| 37 | 1.7 | 67.3 | 16.7 | 9.5 | FPA (BA10) | 100.0 |
| 38 | -26.3 | 66.3 | 17.3 | 9.3 | FPA (BA10) | 88.1 |
| 39 | -49.0 | 45.3 | 14.3 | 9.5 | L-PTr; Broca's area (BA45) | 66.0 |
| 40 | -60.7 | 15.3 | 12.3 | 10.5 | L-PMC and SMA (BA6) | 36.4 |
| 41 | -69.0 | -17.3 | 7.7 | 11.7 | L-STG (BA22) | 78.2 |
| 42 | -69.0 | -47.7 | 7.3 | 11.9 | L-STG (BA22) | 45.2 |
| 43 | 73.0 | -26.3 | -7.7 | 11.8 | R-MTG (BA21) | 91.7 |
| 44 | 65.3 | 3.7 | -5.3 | 11.7 | R-MTG (BA21) | 50.7 |
| 45 | 57.0 | 38.3 | -0.7 | 8.5 | R-PTr (BA45) | 81.5 |
| 46 | 42.0 | 62.0 | 1.0 | 8.7 | FPA (BA10) | 75.5 |
| 47 | 15.7 | 73.0 | 2.3 | 8.6 | FPA (BA10) | 82.9 |
| 48 | -13.3 | 74.0 | 2.7 | 8.8 | FPA (BA10) | 84.1 |
| 49 | -39.3 | 61.7 | 0.7 | 9.0 | FPA (BA10) | 78.3 |
| 50 | -55.0 | 36.7 | -1.3 | 9.0 | L-PTr; Broca's area (BA45) | 82.7 |
| 51 | -64.0 | 0.7 | -6.3 | 11.9 | L-MTG (BA21) | 61.1 |
| 52 | -71.0 | -28.7 | -8.7 | 10.9 | L-MTG (BA21) | 86.3 |

Notes. Abbreviations: dorsolateral prefrontal cortex: DLPFC; frontal eye fields: FEF; frontopolar area: FPA; middle temporal gyrus: MTG; pre-motor cortex: PMC; pars opercularis: POp; pars triangularis: PTr; subcentral area: SCA; supplementary motor area: SMA; supramarginal gyrus: SMG; superior temporal gyrus: STG; primary motor cortex: S1.

**Supplementary Table 3**. Descriptive data of β-values for each channel and each task condition in oxy-Hb

|  |  | English-into-Japanese | | | | |  | Japanese-into-English | | | | |
| --- | --- | --- | --- | --- | --- | --- | --- | --- | --- | --- | --- | --- |
|  |  | high familiairity | |  | low familiarity | |  | high familiairity | |  | low familiarity | |
| CH | *n* | *M* | *SD* |  | *M* | *SD* |  | *M* | *SD* |  | *M* | *SD* |
| 1 | 26 | 0.00 | 0.12 |  | 0.03 | 0.12 |  | 0.03 | 0.13 |  | 0.05 | 0.11 |
| 2 | 26 | -0.03 | 0.19 |  | 0.02 | 0.18 |  | 0.00 | 0.20 |  | 0.04 | 0.18 |
| 3 | 26 | -0.05 | 0.11 |  | 0.00 | 0.09 |  | -0.01 | 0.11 |  | 0.02 | 0.10 |
| 4 | 27 | -0.01 | 0.09 |  | 0.01 | 0.09 |  | 0.01 | 0.08 |  | 0.02 | 0.09 |
| 5 | 30 | 0.01 | 0.06 |  | 0.02 | 0.07 |  | 0.02 | 0.09 |  | 0.02 | 0.07 |
| 6 | 30 | 0.00 | 0.06 |  | 0.01 | 0.08 |  | 0.00 | 0.08 |  | 0.01 | 0.08 |
| 7 | 28 | -0.02 | 0.07 |  | 0.02 | 0.09 |  | 0.00 | 0.08 |  | 0.01 | 0.07 |
| 8 | 28 | 0.00 | 0.07 |  | 0.05 | 0.09 |  | 0.03 | 0.09 |  | 0.05 | 0.09 |
| 9 | 25 | 0.00 | 0.09 |  | 0.02 | 0.09 |  | 0.01 | 0.10 |  | 0.03 | 0.09 |
| 10 | 30 | 0.01 | 0.06 |  | 0.03 | 0.06 |  | 0.02 | 0.07 |  | 0.03 | 0.06 |
| 11 | 28 | -0.01 | 0.11 |  | 0.01 | 0.09 |  | 0.01 | 0.10 |  | 0.02 | 0.08 |
| 12 | 22 | 0.01 | 0.14 |  | 0.04 | 0.13 |  | 0.03 | 0.12 |  | 0.06 | 0.11 |
| 13 | 24 | 0.00 | 0.14 |  | 0.06 | 0.15 |  | 0.05 | 0.16 |  | 0.07 | 0.11 |
| 14 | 27 | -0.01 | 0.09 |  | 0.03 | 0.10 |  | 0.02 | 0.09 |  | 0.04 | 0.08 |
| 15 | 30 | -0.01 | 0.08 |  | 0.01 | 0.08 |  | 0.01 | 0.08 |  | 0.02 | 0.08 |
| 16 | 31 | 0.00 | 0.08 |  | 0.01 | 0.07 |  | 0.01 | 0.07 |  | 0.01 | 0.08 |
| 17 | 30 | 0.00 | 0.08 |  | 0.03 | 0.11 |  | 0.01 | 0.08 |  | 0.03 | 0.09 |
| 18 | 30 | -0.02 | 0.07 |  | 0.03 | 0.08 |  | 0.02 | 0.08 |  | 0.04 | 0.08 |
| 19 | 29 | 0.01 | 0.09 |  | 0.04 | 0.11 |  | 0.02 | 0.11 |  | 0.05 | 0.11 |
| 20 | 30 | -0.01 | 0.08 |  | 0.02 | 0.09 |  | 0.00 | 0.10 |  | 0.02 | 0.08 |
| 21 | 30 | -0.01 | 0.08 |  | 0.02 | 0.07 |  | 0.01 | 0.08 |  | 0.02 | 0.08 |
| 22 | 24 | -0.01 | 0.19 |  | 0.04 | 0.14 |  | 0.03 | 0.16 |  | 0.02 | 0.12 |
| 23 | 22 | -0.03 | 0.23 |  | 0.00 | 0.19 |  | 0.01 | 0.18 |  | 0.00 | 0.21 |
| 24 | 29 | -0.01 | 0.11 |  | 0.03 | 0.10 |  | 0.03 | 0.12 |  | 0.03 | 0.10 |
| 25 | 31 | -0.02 | 0.08 |  | 0.03 | 0.09 |  | 0.00 | 0.09 |  | 0.03 | 0.08 |
| 26 | 31 | 0.00 | 0.08 |  | 0.03 | 0.08 |  | 0.02 | 0.07 |  | 0.04 | 0.07 |
| 27 | 31 | 0.00 | 0.06 |  | 0.02 | 0.07 |  | 0.02 | 0.06 |  | 0.03 | 0.06 |
| 28 | 31 | 0.01 | 0.08 |  | 0.04 | 0.07 |  | 0.03 | 0.08 |  | 0.06 | 0.08 |
| 29 | 30 | -0.01 | 0.10 |  | 0.04 | 0.09 |  | 0.03 | 0.12 |  | 0.05 | 0.13 |
| 30 | 26 | 0.00 | 0.11 |  | 0.01 | 0.15 |  | 0.02 | 0.15 |  | 0.01 | 0.12 |
| 31 | 29 | -0.01 | 0.11 |  | 0.01 | 0.10 |  | 0.00 | 0.13 |  | 0.01 | 0.10 |
| 32 | 27 | -0.01 | 0.13 |  | 0.02 | 0.10 |  | 0.02 | 0.12 |  | 0.01 | 0.10 |
| 33 | 23 | 0.00 | 0.15 |  | 0.06 | 0.18 |  | 0.07 | 0.19 |  | 0.02 | 0.15 |
| 34 | 25 | 0.03 | 0.17 |  | 0.03 | 0.15 |  | 0.05 | 0.19 |  | 0.07 | 0.15 |
| 35 | 29 | -0.02 | 0.10 |  | 0.03 | 0.10 |  | 0.02 | 0.11 |  | 0.04 | 0.08 |
| 36 | 28 | -0.01 | 0.12 |  | 0.04 | 0.12 |  | 0.02 | 0.08 |  | 0.05 | 0.11 |
| 37 | 30 | -0.02 | 0.12 |  | 0.02 | 0.11 |  | 0.01 | 0.09 |  | 0.02 | 0.10 |
| 38 | 30 | 0.03 | 0.11 |  | 0.08 | 0.10 |  | 0.05 | 0.08 |  | 0.08 | 0.10 |
| 39 | 29 | 0.01 | 0.09 |  | 0.07 | 0.10 |  | 0.05 | 0.11 |  | 0.08 | 0.12 |
| 40 | 24 | 0.01 | 0.13 |  | 0.05 | 0.16 |  | 0.06 | 0.15 |  | 0.03 | 0.13 |
| 41 | 24 | -0.04 | 0.14 |  | -0.02 | 0.14 |  | 0.01 | 0.17 |  | -0.02 | 0.15 |
| 42 | 29 | 0.00 | 0.13 |  | 0.03 | 0.12 |  | 0.02 | 0.15 |  | 0.02 | 0.15 |
| 43 | 27 | -0.01 | 0.14 |  | 0.03 | 0.13 |  | 0.04 | 0.18 |  | 0.01 | 0.14 |
| 44 | 26 | 0.01 | 0.19 |  | 0.03 | 0.17 |  | 0.06 | 0.22 |  | 0.05 | 0.16 |
| 45 | 28 | -0.01 | 0.15 |  | 0.03 | 0.16 |  | 0.05 | 0.14 |  | 0.05 | 0.14 |
| 46 | 28 | 0.00 | 0.15 |  | 0.06 | 0.17 |  | 0.04 | 0.09 |  | 0.06 | 0.12 |
| 47 | 26 | -0.02 | 0.14 |  | 0.04 | 0.16 |  | 0.01 | 0.09 |  | 0.03 | 0.09 |
| 48 | 29 | -0.01 | 0.15 |  | 0.06 | 0.14 |  | 0.03 | 0.11 |  | 0.05 | 0.11 |
| 49 | 28 | 0.01 | 0.14 |  | 0.09 | 0.15 |  | 0.05 | 0.10 |  | 0.07 | 0.11 |
| 50 | 26 | -0.01 | 0.11 |  | 0.07 | 0.13 |  | 0.05 | 0.12 |  | 0.06 | 0.16 |
| 51 | 24 | -0.01 | 0.18 |  | 0.05 | 0.20 |  | 0.05 | 0.23 |  | 0.01 | 0.22 |
| 52 | 26 | -0.02 | 0.14 |  | 0.01 | 0.13 |  | 0.03 | 0.17 |  | 0.00 | 0.19 |

**Supplementary Table 4**. Descriptive data of β-values for each channel and each task condition in deoxy-Hb

|  |  | English-into-Japanese | | | | |  | Japanese-into-English | | | | |
| --- | --- | --- | --- | --- | --- | --- | --- | --- | --- | --- | --- | --- |
|  |  | high familiairity | |  | low familiarity | |  | high familiairity | |  | low familiarity | |
| CH | *n* | *M* | *SD* |  | *M* | *SD* |  | *M* | *SD* |  | *M* | *SD* |
| 1 | 27 | -0.01 | 0.13 |  | -0.02 | 0.17 |  | -0.02 | 0.15 |  | -0.02 | 0.16 |
| 2 | 27 | -0.01 | 0.08 |  | -0.01 | 0.10 |  | -0.03 | 0.10 |  | -0.02 | 0.09 |
| 3 | 26 | -0.01 | 0.07 |  | -0.01 | 0.07 |  | -0.02 | 0.08 |  | -0.01 | 0.08 |
| 4 | 27 | -0.01 | 0.06 |  | 0.01 | 0.05 |  | 0.00 | 0.04 |  | 0.01 | 0.04 |
| 5 | 30 | 0.01 | 0.04 |  | 0.01 | 0.04 |  | 0.01 | 0.03 |  | 0.01 | 0.04 |
| 6 | 31 | 0.01 | 0.04 |  | 0.01 | 0.04 |  | 0.01 | 0.05 |  | 0.01 | 0.03 |
| 7 | 29 | 0.01 | 0.07 |  | 0.01 | 0.04 |  | 0.01 | 0.06 |  | 0.00 | 0.07 |
| 8 | 29 | -0.01 | 0.09 |  | -0.01 | 0.11 |  | -0.02 | 0.11 |  | 0.00 | 0.08 |
| 9 | 26 | -0.03 | 0.08 |  | -0.03 | 0.15 |  | -0.04 | 0.10 |  | -0.01 | 0.08 |
| 10 | 30 | 0.01 | 0.03 |  | 0.02 | 0.03 |  | 0.01 | 0.03 |  | 0.02 | 0.04 |
| 11 | 29 | 0.00 | 0.03 |  | 0.00 | 0.05 |  | 0.01 | 0.05 |  | 0.00 | 0.04 |
| 12 | 23 | 0.01 | 0.08 |  | 0.03 | 0.09 |  | 0.03 | 0.08 |  | 0.02 | 0.08 |
| 13 | 24 | -0.02 | 0.08 |  | 0.00 | 0.07 |  | 0.00 | 0.08 |  | 0.00 | 0.07 |
| 14 | 27 | -0.01 | 0.04 |  | 0.00 | 0.04 |  | -0.01 | 0.03 |  | 0.00 | 0.02 |
| 15 | 31 | 0.01 | 0.05 |  | 0.02 | 0.04 |  | 0.00 | 0.05 |  | 0.01 | 0.04 |
| 16 | 32 | 0.00 | 0.04 |  | 0.02 | 0.06 |  | 0.01 | 0.05 |  | 0.02 | 0.06 |
| 17 | 31 | 0.01 | 0.04 |  | 0.03 | 0.08 |  | 0.02 | 0.05 |  | 0.02 | 0.04 |
| 18 | 31 | 0.01 | 0.04 |  | 0.02 | 0.04 |  | 0.02 | 0.04 |  | 0.02 | 0.03 |
| 19 | 30 | -0.02 | 0.07 |  | -0.01 | 0.10 |  | -0.03 | 0.08 |  | -0.02 | 0.07 |
| 20 | 30 | -0.01 | 0.05 |  | 0.00 | 0.05 |  | 0.00 | 0.04 |  | 0.00 | 0.05 |
| 21 | 31 | -0.01 | 0.03 |  | 0.00 | 0.02 |  | 0.00 | 0.03 |  | 0.00 | 0.04 |
| 22 | 25 | 0.00 | 0.08 |  | 0.02 | 0.07 |  | 0.03 | 0.08 |  | 0.02 | 0.08 |
| 23 | 23 | -0.02 | 0.12 |  | 0.02 | 0.11 |  | 0.03 | 0.10 |  | 0.01 | 0.11 |
| 24 | 30 | 0.00 | 0.05 |  | 0.02 | 0.07 |  | 0.01 | 0.04 |  | 0.01 | 0.05 |
| 25 | 32 | 0.00 | 0.04 |  | 0.01 | 0.04 |  | 0.00 | 0.04 |  | 0.02 | 0.04 |
| 26 | 32 | 0.00 | 0.03 |  | 0.01 | 0.03 |  | 0.00 | 0.03 |  | 0.01 | 0.03 |
| 27 | 32 | 0.00 | 0.03 |  | 0.01 | 0.03 |  | 0.00 | 0.03 |  | 0.00 | 0.03 |
| 28 | 32 | 0.00 | 0.03 |  | 0.01 | 0.03 |  | 0.00 | 0.03 |  | 0.02 | 0.03 |
| 29 | 31 | 0.01 | 0.05 |  | 0.03 | 0.04 |  | 0.02 | 0.05 |  | 0.02 | 0.05 |
| 30 | 27 | -0.02 | 0.09 |  | 0.00 | 0.10 |  | 0.00 | 0.08 |  | -0.02 | 0.08 |
| 31 | 30 | -0.02 | 0.06 |  | 0.00 | 0.07 |  | 0.00 | 0.05 |  | 0.00 | 0.06 |
| 32 | 28 | 0.01 | 0.05 |  | 0.01 | 0.07 |  | 0.02 | 0.06 |  | 0.02 | 0.07 |
| 33 | 24 | 0.00 | 0.09 |  | 0.02 | 0.09 |  | 0.01 | 0.11 |  | 0.02 | 0.08 |
| 34 | 26 | 0.00 | 0.11 |  | 0.02 | 0.09 |  | 0.03 | 0.12 |  | 0.00 | 0.11 |
| 35 | 30 | 0.00 | 0.04 |  | 0.01 | 0.04 |  | 0.00 | 0.04 |  | 0.01 | 0.04 |
| 36 | 29 | -0.01 | 0.04 |  | 0.01 | 0.03 |  | 0.00 | 0.04 |  | 0.01 | 0.04 |
| 37 | 31 | 0.01 | 0.03 |  | 0.02 | 0.04 |  | 0.01 | 0.04 |  | 0.02 | 0.04 |
| 38 | 31 | 0.01 | 0.05 |  | 0.03 | 0.04 |  | 0.02 | 0.05 |  | 0.02 | 0.05 |
| 39 | 30 | 0.00 | 0.05 |  | 0.01 | 0.04 |  | 0.01 | 0.04 |  | 0.00 | 0.05 |
| 40 | 24 | 0.00 | 0.08 |  | -0.01 | 0.10 |  | 0.02 | 0.12 |  | 0.01 | 0.07 |
| 41 | 25 | -0.03 | 0.10 |  | -0.01 | 0.11 |  | 0.02 | 0.09 |  | 0.00 | 0.10 |
| 42 | 30 | 0.00 | 0.05 |  | 0.02 | 0.05 |  | 0.02 | 0.04 |  | 0.02 | 0.06 |
| 43 | 28 | 0.00 | 0.07 |  | 0.02 | 0.07 |  | 0.02 | 0.09 |  | 0.03 | 0.07 |
| 44 | 27 | 0.02 | 0.11 |  | 0.02 | 0.10 |  | 0.03 | 0.13 |  | 0.02 | 0.12 |
| 45 | 29 | -0.01 | 0.08 |  | -0.01 | 0.09 |  | 0.01 | 0.07 |  | -0.01 | 0.11 |
| 46 | 29 | -0.01 | 0.06 |  | 0.01 | 0.05 |  | 0.00 | 0.06 |  | 0.01 | 0.05 |
| 47 | 27 | 0.00 | 0.04 |  | 0.02 | 0.03 |  | 0.01 | 0.02 |  | 0.02 | 0.03 |
| 48 | 30 | 0.00 | 0.05 |  | 0.02 | 0.04 |  | 0.01 | 0.04 |  | 0.01 | 0.05 |
| 49 | 29 | 0.01 | 0.05 |  | 0.03 | 0.06 |  | 0.03 | 0.06 |  | 0.03 | 0.05 |
| 50 | 27 | 0.00 | 0.09 |  | 0.00 | 0.08 |  | 0.01 | 0.10 |  | 0.00 | 0.08 |
| 51 | 25 | 0.01 | 0.11 |  | 0.03 | 0.11 |  | 0.03 | 0.14 |  | 0.04 | 0.09 |
| 52 | 27 | 0.00 | 0.08 |  | 0.02 | 0.10 |  | 0.03 | 0.07 |  | 0.03 | 0.07 |

**Supplementary Table 5**. Results of one-sample *t*-tests (vs.0) in oxy-Hb for each channel and each task condition.

|  | English-into-Japanese | | | | | | |  | Japanese-into-English | | | | | | |
| --- | --- | --- | --- | --- | --- | --- | --- | --- | --- | --- | --- | --- | --- | --- | --- |
|  | high familiairity | | |  | low familiarity | | |  | high familiairity | | |  | low familiarity | | |
| CH | *t* | *p* | *d* |  | *t* | *p* | *d* |  | *t* | *p* | *d* |  | *t* | *p* | *d* |
| 1 | 0.08 | 1.00 | 0.02 |  | 1.34 | 1.00 | 0.26 |  | 1.27 | 1.00 | 0.25 |  | 2.04 | 1.00 | 0.40 |
| 2 | -0.85 | 1.00 | -0.17 |  | 0.46 | 1.00 | 0.09 |  | -0.01 | 1.00 | 0.00 |  | 1.17 | 1.00 | 0.23 |
| 3 | -2.23 | 0.86 | -0.44 |  | -0.04 | 1.00 | -0.01 |  | -0.70 | 1.00 | -0.14 |  | 1.04 | 1.00 | 0.20 |
| 4 | -0.36 | 1.00 | -0.07 |  | 0.47 | 1.00 | 0.09 |  | 0.82 | 1.00 | 0.16 |  | 1.29 | 1.00 | 0.25 |
| 5 | 1.04 | 1.00 | 0.19 |  | 1.96 | 1.00 | 0.36 |  | 1.03 | 1.00 | 0.19 |  | 1.67 | 1.00 | 0.31 |
| 6 | 0.21 | 1.00 | 0.04 |  | 0.94 | 1.00 | 0.17 |  | 0.19 | 1.00 | 0.03 |  | 0.73 | 1.00 | 0.13 |
| 7 | -1.23 | 1.00 | -0.23 |  | 1.08 | 1.00 | 0.20 |  | -0.23 | 1.00 | -0.04 |  | 0.53 | 1.00 | 0.10 |
| 8 | 0.28 | 1.00 | 0.05 |  | 2.99 | 0.13 | 0.57 |  | 1.69 | 1.00 | 0.32 |  | 2.91 | 0.17 | 0.55 |
| 9 | -0.18 | 1.00 | -0.04 |  | 0.87 | 1.00 | 0.17 |  | 0.27 | 1.00 | 0.05 |  | 1.83 | 1.00 | 0.37 |
| 10 | 1.08 | 1.00 | 0.20 |  | 2.62 | 0.32 | 0.48 |  | 1.15 | 1.00 | 0.21 |  | 2.71 | 0.26 | 0.50 |
| 11 | -0.48 | 1.00 | -0.09 |  | 0.77 | 1.00 | 0.15 |  | 0.62 | 1.00 | 0.12 |  | 1.15 | 1.00 | 0.22 |
| 12 | 0.40 | 1.00 | 0.08 |  | 1.39 | 1.00 | 0.30 |  | 1.24 | 1.00 | 0.27 |  | 2.85 | 0.23 | 0.61 |
| 13 | -0.17 | 1.00 | -0.03 |  | 1.76 | 1.00 | 0.36 |  | 1.70 | 1.00 | 0.35 |  | 2.98 | 0.16 | 0.61 |
| 14 | -0.56 | 1.00 | -0.11 |  | 1.65 | 1.00 | 0.32 |  | 1.15 | 1.00 | 0.22 |  | 2.53 | 0.42 | 0.49 |
| 15 | -0.91 | 1.00 | -0.17 |  | 0.90 | 1.00 | 0.16 |  | 0.51 | 1.00 | 0.09 |  | 1.50 | 1.00 | 0.27 |
| 16 | -0.18 | 1.00 | -0.03 |  | 0.64 | 1.00 | 0.12 |  | 0.52 | 1.00 | 0.09 |  | 0.94 | 1.00 | 0.17 |
| 17 | 0.22 | 1.00 | 0.04 |  | 1.36 | 1.00 | 0.25 |  | 0.93 | 1.00 | 0.17 |  | 1.60 | 1.00 | 0.29 |
| 18 | -1.33 | 1.00 | -0.24 |  | 1.91 | 1.00 | 0.35 |  | 1.13 | 1.00 | 0.21 |  | 2.62 | 0.33 | 0.48 |
| 19 | 0.61 | 1.00 | 0.11 |  | 2.13 | 0.96 | 0.40 |  | 1.21 | 1.00 | 0.23 |  | 2.18 | 0.90 | 0.41 |
| 20 | -0.82 | 1.00 | -0.15 |  | 1.02 | 1.00 | 0.19 |  | 0.16 | 1.00 | 0.03 |  | 1.01 | 1.00 | 0.18 |
| 21 | -0.46 | 1.00 | -0.08 |  | 1.53 | 1.00 | 0.28 |  | 0.55 | 1.00 | 0.10 |  | 1.52 | 1.00 | 0.28 |
| 22 | -0.27 | 1.00 | -0.05 |  | 1.40 | 1.00 | 0.29 |  | 0.80 | 1.00 | 0.16 |  | 0.97 | 1.00 | 0.20 |
| 23 | -0.67 | 1.00 | -0.14 |  | -0.10 | 1.00 | -0.02 |  | 0.19 | 1.00 | 0.04 |  | 0.08 | 1.00 | 0.02 |
| 24 | -0.57 | 1.00 | -0.11 |  | 1.44 | 1.00 | 0.27 |  | 1.50 | 1.00 | 0.28 |  | 1.75 | 1.00 | 0.33 |
| 25 | -1.40 | 1.00 | -0.25 |  | 1.71 | 1.00 | 0.31 |  | 0.21 | 1.00 | 0.04 |  | 2.27 | 0.73 | 0.41 |
| 26 | 0.06 | 1.00 | 0.01 |  | 2.06 | 1.00 | 0.37 |  | 1.91 | 1.00 | 0.34 |  | 3.22 | 0.07 | 0.58 |
| 27 | -0.24 | 1.00 | -0.04 |  | 1.62 | 1.00 | 0.29 |  | 1.61 | 1.00 | 0.29 |  | 2.85 | 0.19 | 0.51 |
| 28 | 0.40 | 1.00 | 0.07 |  | 3.06 | 0.10 | 0.55 |  | 2.25 | 0.79 | 0.40 |  | 3.79 | 0.02 | 0.68 |
| 29 | -0.43 | 1.00 | -0.08 |  | 2.16 | 0.89 | 0.39 |  | 1.49 | 1.00 | 0.27 |  | 2.11 | 1.00 | 0.38 |
| 30 | 0.00 | 1.00 | 0.00 |  | 0.40 | 1.00 | 0.08 |  | 0.55 | 1.00 | 0.11 |  | 0.22 | 1.00 | 0.04 |
| 31 | -0.73 | 1.00 | -0.14 |  | 0.65 | 1.00 | 0.12 |  | 0.09 | 1.00 | 0.02 |  | 0.61 | 1.00 | 0.11 |
| 32 | -0.56 | 1.00 | -0.11 |  | 0.88 | 1.00 | 0.17 |  | 0.94 | 1.00 | 0.18 |  | 0.33 | 1.00 | 0.06 |
| 33 | -0.07 | 1.00 | -0.01 |  | 1.71 | 1.00 | 0.36 |  | 1.69 | 1.00 | 0.35 |  | 0.74 | 1.00 | 0.15 |
| 34 | 0.77 | 1.00 | 0.15 |  | 0.85 | 1.00 | 0.17 |  | 1.33 | 1.00 | 0.27 |  | 2.35 | 0.66 | 0.47 |
| 35 | -0.99 | 1.00 | -0.18 |  | 1.37 | 1.00 | 0.25 |  | 1.08 | 1.00 | 0.20 |  | 2.83 | 0.20 | 0.53 |
| 36 | -0.41 | 1.00 | -0.08 |  | 1.85 | 1.00 | 0.35 |  | 1.10 | 1.00 | 0.21 |  | 2.47 | 0.48 | 0.47 |
| 37 | -0.95 | 1.00 | -0.17 |  | 1.15 | 1.00 | 0.21 |  | 0.74 | 1.00 | 0.14 |  | 1.23 | 1.00 | 0.22 |
| 38 | 1.41 | 1.00 | 0.26 |  | 4.12 | 0.01 | 0.75 |  | 3.60 | 0.03 | 0.66 |  | 4.38 | 0.00 | 0.80 |
| 39 | 0.76 | 1.00 | 0.14 |  | 3.79 | 0.02 | 0.70 |  | 2.60 | 0.36 | 0.48 |  | 3.69 | 0.02 | 0.69 |
| 40 | 0.38 | 1.00 | 0.08 |  | 1.64 | 1.00 | 0.34 |  | 1.92 | 1.00 | 0.39 |  | 1.25 | 1.00 | 0.26 |
| 41 | -1.45 | 1.00 | -0.30 |  | -0.53 | 1.00 | -0.11 |  | 0.39 | 1.00 | 0.08 |  | -0.80 | 1.00 | -0.16 |
| 42 | -0.19 | 1.00 | -0.04 |  | 1.37 | 1.00 | 0.25 |  | 0.85 | 1.00 | 0.16 |  | 0.55 | 1.00 | 0.10 |
| 43 | -0.30 | 1.00 | -0.06 |  | 1.26 | 1.00 | 0.24 |  | 1.25 | 1.00 | 0.24 |  | 0.31 | 1.00 | 0.06 |
| 44 | 0.22 | 1.00 | 0.04 |  | 1.01 | 1.00 | 0.20 |  | 1.31 | 1.00 | 0.26 |  | 1.49 | 1.00 | 0.29 |
| 45 | -0.29 | 1.00 | -0.06 |  | 0.93 | 1.00 | 0.18 |  | 1.91 | 1.00 | 0.36 |  | 1.77 | 1.00 | 0.33 |
| 46 | 0.05 | 1.00 | 0.01 |  | 2.00 | 1.00 | 0.38 |  | 2.28 | 0.76 | 0.43 |  | 2.82 | 0.21 | 0.53 |
| 47 | -0.81 | 1.00 | -0.16 |  | 1.26 | 1.00 | 0.25 |  | 0.47 | 1.00 | 0.09 |  | 1.37 | 1.00 | 0.27 |
| 48 | -0.24 | 1.00 | -0.04 |  | 2.31 | 0.64 | 0.43 |  | 1.70 | 1.00 | 0.32 |  | 2.46 | 0.48 | 0.46 |
| 49 | 0.45 | 1.00 | 0.09 |  | 2.99 | 0.13 | 0.56 |  | 2.52 | 0.44 | 0.48 |  | 3.15 | 0.09 | 0.60 |
| 50 | -0.32 | 1.00 | -0.06 |  | 2.63 | 0.33 | 0.52 |  | 2.08 | 1.00 | 0.41 |  | 1.92 | 1.00 | 0.38 |
| 51 | -0.37 | 1.00 | -0.08 |  | 1.25 | 1.00 | 0.26 |  | 1.17 | 1.00 | 0.24 |  | 0.14 | 1.00 | 0.03 |
| 52 | -0.86 | 1.00 | -0.17 |  | 0.50 | 1.00 | 0.10 |  | 0.94 | 1.00 | 0.18 |  | -0.11 | 1.00 | -0.02 |

Notes. *p*-values were corrected using M_eff_ correction and capped at 1.00.

**Supplementary Table 6.** Results of one-sample *t*-tests (vs.0) in deoxy-Hb for each channel and each task condition.

|  | English-into-Japanese | | | | | | |  | Japanese-into-English | | | | | | |
| --- | --- | --- | --- | --- | --- | --- | --- | --- | --- | --- | --- | --- | --- | --- | --- |
|  | high familiairity | | |  | low familiarity | | |  | high familiairity | | |  | low familiarity | | |
| CH | *t* | *p* | *d* |  | *t* | *p* | *d* |  | *t* | *p* | *d* |  | *t* | *p* | *d* |
| 1 | -0.57 | 1.00 | -0.11 |  | -0.53 | 1.00 | -0.10 |  | -0.79 | 1.00 | -0.15 |  | -0.75 | 1.00 | -0.14 |
| 2 | -0.83 | 1.00 | -0.16 |  | -0.53 | 1.00 | -0.10 |  | -1.39 | 1.00 | -0.27 |  | -1.39 | 1.00 | -0.27 |
| 3 | -1.04 | 1.00 | -0.20 |  | -0.83 | 1.00 | -0.16 |  | -1.26 | 1.00 | -0.25 |  | -0.44 | 1.00 | -0.09 |
| 4 | -0.98 | 1.00 | -0.19 |  | 1.34 | 1.00 | 0.26 |  | -0.45 | 1.00 | -0.09 |  | 1.40 | 1.00 | 0.27 |
| 5 | 0.79 | 1.00 | 0.14 |  | 1.86 | 1.00 | 0.34 |  | 0.90 | 1.00 | 0.16 |  | 1.52 | 1.00 | 0.28 |
| 6 | 1.78 | 1.00 | 0.32 |  | 2.06 | 1.00 | 0.37 |  | 1.67 | 1.00 | 0.30 |  | 1.57 | 1.00 | 0.28 |
| 7 | 0.72 | 1.00 | 0.13 |  | 1.56 | 1.00 | 0.29 |  | 1.06 | 1.00 | 0.20 |  | 0.28 | 1.00 | 0.05 |
| 8 | -0.53 | 1.00 | -0.10 |  | -0.59 | 1.00 | -0.11 |  | -1.04 | 1.00 | -0.19 |  | -0.26 | 1.00 | -0.05 |
| 9 | -1.67 | 1.00 | -0.33 |  | -1.03 | 1.00 | -0.20 |  | -1.89 | 1.00 | -0.37 |  | -0.81 | 1.00 | -0.16 |
| 10 | 2.38 | 0.69 | 0.43 |  | 2.45 | 0.50 | 0.45 |  | 1.20 | 1.00 | 0.22 |  | 2.52 | 0.49 | 0.46 |
| 11 | -0.25 | 1.00 | -0.05 |  | -0.17 | 1.00 | -0.03 |  | 0.59 | 1.00 | 0.11 |  | 0.22 | 1.00 | 0.04 |
| 12 | 0.55 | 1.00 | 0.12 |  | 1.37 | 1.00 | 0.29 |  | 1.72 | 1.00 | 0.36 |  | 0.98 | 1.00 | 0.20 |
| 13 | -1.09 | 1.00 | -0.22 |  | -0.08 | 1.00 | -0.02 |  | -0.09 | 1.00 | -0.02 |  | -0.13 | 1.00 | -0.03 |
| 14 | -1.29 | 1.00 | -0.25 |  | 0.37 | 1.00 | 0.07 |  | -1.57 | 1.00 | -0.30 |  | -0.42 | 1.00 | -0.08 |
| 15 | 0.74 | 1.00 | 0.13 |  | 1.94 | 1.00 | 0.35 |  | 0.47 | 1.00 | 0.09 |  | 1.37 | 1.00 | 0.25 |
| 16 | 0.42 | 1.00 | 0.07 |  | 2.12 | 1.00 | 0.37 |  | 0.75 | 1.00 | 0.13 |  | 1.86 | 1.00 | 0.33 |
| 17 | 1.84 | 1.00 | 0.33 |  | 2.14 | 1.00 | 0.38 |  | 1.75 | 1.00 | 0.31 |  | 2.95 | 0.17 | 0.53 |
| 18 | 1.83 | 1.00 | 0.33 |  | 2.65 | 0.31 | 0.48 |  | 2.04 | 1.00 | 0.37 |  | 3.07 | 0.13 | 0.55 |
| 19 | -2.01 | 1.00 | -0.37 |  | -0.71 | 1.00 | -0.13 |  | -1.78 | 1.00 | -0.32 |  | -1.64 | 1.00 | -0.30 |
| 20 | -1.67 | 1.00 | -0.30 |  | 0.19 | 1.00 | 0.04 |  | 0.05 | 1.00 | 0.01 |  | -0.25 | 1.00 | -0.05 |
| 21 | -0.99 | 1.00 | -0.18 |  | 0.48 | 1.00 | 0.09 |  | -0.80 | 1.00 | -0.14 |  | 0.56 | 1.00 | 0.10 |
| 22 | 0.26 | 1.00 | 0.05 |  | 1.55 | 1.00 | 0.31 |  | 1.77 | 1.00 | 0.35 |  | 0.99 | 1.00 | 0.20 |
| 23 | -0.60 | 1.00 | -0.12 |  | 0.83 | 1.00 | 0.17 |  | 1.22 | 1.00 | 0.26 |  | 0.48 | 1.00 | 0.10 |
| 24 | -0.44 | 1.00 | -0.08 |  | 1.47 | 1.00 | 0.27 |  | 1.99 | 1.00 | 0.36 |  | 1.48 | 1.00 | 0.27 |
| 25 | -0.10 | 1.00 | -0.02 |  | 1.84 | 1.00 | 0.33 |  | 0.26 | 1.00 | 0.05 |  | 2.40 | 0.64 | 0.42 |
| 26 | -0.25 | 1.00 | -0.04 |  | 1.75 | 1.00 | 0.31 |  | 0.58 | 1.00 | 0.10 |  | 1.89 | 1.00 | 0.33 |
| 27 | -0.78 | 1.00 | -0.14 |  | 1.13 | 1.00 | 0.20 |  | -0.98 | 1.00 | -0.17 |  | 0.58 | 1.00 | 0.10 |
| 28 | 0.77 | 1.00 | 0.14 |  | 2.04 | 1.00 | 0.36 |  | 0.69 | 1.00 | 0.12 |  | 3.66 | 0.03 | 0.65 |
| 29 | 0.96 | 1.00 | 0.17 |  | 3.88 | 0.01 | 0.70 |  | 2.45 | 0.54 | 0.44 |  | 2.13 | 1.00 | 0.38 |
| 30 | -1.40 | 1.00 | -0.27 |  | -0.21 | 1.00 | -0.04 |  | 0.10 | 1.00 | 0.02 |  | -1.20 | 1.00 | -0.23 |
| 31 | -1.60 | 1.00 | -0.29 |  | 0.04 | 1.00 | 0.01 |  | 0.42 | 1.00 | 0.08 |  | -0.36 | 1.00 | -0.07 |
| 32 | 0.69 | 1.00 | 0.13 |  | 0.81 | 1.00 | 0.15 |  | 2.22 | 0.92 | 0.42 |  | 1.50 | 1.00 | 0.28 |
| 33 | -0.05 | 1.00 | -0.01 |  | 0.85 | 1.00 | 0.17 |  | 0.38 | 1.00 | 0.08 |  | 1.25 | 1.00 | 0.25 |
| 34 | -0.09 | 1.00 | -0.02 |  | 0.87 | 1.00 | 0.17 |  | 1.03 | 1.00 | 0.20 |  | 0.04 | 1.00 | 0.01 |
| 35 | -0.65 | 1.00 | -0.12 |  | 0.88 | 1.00 | 0.16 |  | 0.54 | 1.00 | 0.10 |  | 1.83 | 1.00 | 0.33 |
| 36 | -0.86 | 1.00 | -0.16 |  | 1.92 | 1.00 | 0.36 |  | -0.18 | 1.00 | -0.03 |  | 0.95 | 1.00 | 0.18 |
| 37 | 0.84 | 1.00 | 0.15 |  | 3.03 | 0.12 | 0.54 |  | 2.11 | 1.00 | 0.38 |  | 3.04 | 0.14 | 0.55 |
| 38 | 1.56 | 1.00 | 0.28 |  | 4.23 | 0.00 | 0.76 |  | 2.34 | 0.69 | 0.42 |  | 2.13 | 1.00 | 0.38 |
| 39 | 0.23 | 1.00 | 0.04 |  | 1.27 | 1.00 | 0.23 |  | 0.68 | 1.00 | 0.12 |  | 0.48 | 1.00 | 0.09 |
| 40 | -0.15 | 1.00 | -0.03 |  | -0.27 | 1.00 | -0.05 |  | 0.69 | 1.00 | 0.14 |  | 0.92 | 1.00 | 0.19 |
| 41 | -1.35 | 1.00 | -0.27 |  | -0.31 | 1.00 | -0.06 |  | 1.08 | 1.00 | 0.22 |  | 0.06 | 1.00 | 0.01 |
| 42 | -0.07 | 1.00 | -0.01 |  | 1.94 | 1.00 | 0.35 |  | 2.37 | 0.66 | 0.43 |  | 1.64 | 1.00 | 0.30 |
| 43 | 0.39 | 1.00 | 0.07 |  | 1.31 | 1.00 | 0.25 |  | 1.22 | 1.00 | 0.23 |  | 2.41 | 0.64 | 0.46 |
| 44 | 0.97 | 1.00 | 0.19 |  | 1.17 | 1.00 | 0.23 |  | 1.11 | 1.00 | 0.21 |  | 0.81 | 1.00 | 0.16 |
| 45 | -0.78 | 1.00 | -0.14 |  | -0.34 | 1.00 | -0.06 |  | 0.47 | 1.00 | 0.09 |  | -0.44 | 1.00 | -0.08 |
| 46 | -1.00 | 1.00 | -0.18 |  | 0.88 | 1.00 | 0.16 |  | -0.13 | 1.00 | -0.02 |  | 1.25 | 1.00 | 0.23 |
| 47 | 0.57 | 1.00 | 0.11 |  | 2.68 | 0.31 | 0.52 |  | 2.46 | 0.55 | 0.47 |  | 3.55 | 0.04 | 0.68 |
| 48 | 0.01 | 1.00 | 0.00 |  | 2.89 | 0.18 | 0.53 |  | 1.47 | 1.00 | 0.27 |  | 1.29 | 1.00 | 0.23 |
| 49 | 1.51 | 1.00 | 0.28 |  | 2.81 | 0.22 | 0.52 |  | 2.60 | 0.39 | 0.48 |  | 2.86 | 0.22 | 0.53 |
| 50 | 0.00 | 1.00 | 0.00 |  | 0.30 | 1.00 | 0.06 |  | 0.61 | 1.00 | 0.12 |  | 0.31 | 1.00 | 0.06 |
| 51 | 0.40 | 1.00 | 0.08 |  | 1.27 | 1.00 | 0.25 |  | 1.20 | 1.00 | 0.24 |  | 2.11 | 1.00 | 0.42 |
| 52 | 0.27 | 1.00 | 0.05 |  | 1.21 | 1.00 | 0.23 |  | 2.40 | 0.63 | 0.46 |  | 2.37 | 0.71 | 0.46 |

Notes. *p*-values were corrected using M_eff_ correction and capped at 1.00.

**Supplementary Fig. 1.** Block-averaged activation of each channel in the English-to-Japanese (high familiarity) condition.

**Supplementary Fig. 2.** Block-averaged activation of each channel in the English-to-Japanese (low familiarity) condition.

**Supplementary Fig. 3.** Block-averaged activation of each channel in the Japanese-to-English (high familiarity) condition.

**Supplementary Fig 4.** Block-averaged activation of each channel in the Japanese-to-English (low familiarity) condition.
